# Stepwise lactate kinetics in critically ill patients: prognostic, influencing factors, and clinical phenotype

**DOI:** 10.1186/s12871-021-01293-x

**Published:** 2021-03-19

**Authors:** Bo Tang, Longxiang Su, Dongkai Li, Ye Wang, Qianqian Liu, Guangliang Shan, Yun Long, Dawei Liu, Xiang Zhou

**Affiliations:** 1 Department of Critical Care Medicine, Peking Union Medical College Hospital, Chinese Academy of Medical Science and Peking Union Medical College, Beijing, 100730 China; 2 China & State Key Laboratory of Complex Severe and Rare Diseases, Peking Union Medical College Hospital, Chinese Academy of Medical Science and Peking Union Medical College, Beijing, 100730 China; 3 Department of Epidemiology and Statistics, Institute of Basic Medical Sciences, Chinese Academy of Medical Sciences, School of Basic Medicine, Peking Union Medical College, Beijing, 100730 China; 4 Chinese Center for Disease Control and prevention, Beijing, 100050 China

**Keywords:** Hyperlactatemia, Lactate kinetics, Clinical phenotype

## Abstract

**Background:**

To investigate the optimal target e of lactate kinetics at different time during the resuscitation, the factors that influence whether the kinetics achieve the goals, and the clinical implications of different clinical phenotypes.

**Methods:**

Patients with hyperlactatemia between May 1, 2013 and December 31, 2018 were retrospectively analyzed. Demographic data, basic organ function, hemodynamic parameters at ICU admission (T0) and at 6 h, 12 h, 24 h, 48 h, and 72 h, arterial blood lactate and blood glucose levels, cumulative clinical treatment conditions at different time points and final patient outcomes were collected.

**Results:**

A total of 3298 patients were enrolled, and the mortality rate was 12.2%. The cutoff values of lactate kinetics for prognosis at 6 h, 12 h, 24 h, 48 h, and 72 h were 21%, 40%, 57%, 66%, and 72%. The APACHE II score, SOFA score, heart rate (HR), and blood glucose were risk factors that correlated with whether the lactate kinetics attained the target goal. Based on the pattens of the lactate kinetics, eight clinical phenotypes were proposed. The odds ratios of death for clinical phenotypes VIII, IV, and II were 4.39, 4.2, and 5.27-fold of those of clinical phenotype I, respectively.

**Conclusion:**

Stepwise recovery of lactate kinetics is an important resuscitation target for patients with hyperlactatemia. The APACHE II score, SOFA score, HR, and blood glucose were independent risk factors that influenced achievement of lactate kinetic targets. The cinical phenotypes of stepwise lactate kinetics are closely related to the prognosis.

**Supplementary Information:**

The online version contains supplementary material available at 10.1186/s12871-021-01293-x.

## Background

Hyperlactatemia is a presentation of common homeostasis disorders in critically ill patients and is closely associated with infection, stress, tissue ischemia and hypoxia, and organ dysfunction. Recent studies have indicated that elevation of blood lactate is still an independent risk factor for the intensive care unit (ICU)/hospital mortality rate of critically ill patients [1–4]. Based on this information, further studies showed that blood lactate dynamics, i.e., lactate kinetics, were more meaningful for disease risk stratification under different pathophysiological conditions [5] and were more closely associated with prognosis [6] than lactate absolute values.

Although lactate kinetics have stronger implications regarding clinical guidance than the lactate absolute value, unfortunately, in results from previous studies, the time range of lactate kinetics and the metabolism cutoff values are not consistent. For example, in a sepsis-related study, the lactate kinetics goal at 6 h was 10–34%, which is a large range [7]. As a dynamic indicator, lactate kinetics can not only be used for monitoring but also, more importantly, guide clinical treatment. Many previous studies have confirmed the influence of lactate kinetics on resuscitation [8, 9]. Our previous studies also showed that compared to central venous oxygen saturation (ScvO_2_)-oriented hemodynamic therapy, lactate kinetics-oriented therapy could reduce the mortality of patients with sepsis-associated hyperlactatemia [10].This study further explored the cutoff values for lactate kinetics at different time points and their influence on the mortality rate, analyzed the factors that influencethe achievement of target lactate kinetics, and proposed the significance of different clinical phenotypes of stepwise lactate kinetics.

## Methods

### Patient sample

By examining the administrative database of Peking Union Medical College Hospital, patients with hyperlactatemia (arterial blood lactate≥4.0 mmol/L) who were hospitalized and treated in the ICU of Peking Union Medical College Hospital between May 1, 2013 and December 31, 2018 were retrospectively analyzed. The Institutional Research and Ethics Committee of Peking Union Medical College Hospital approved this study using human subjects.

### Data collection

Arterial blood samples were collected and measured using an ABL blood gas analyzer (ABL90 FLEX, radiometer medical, Copenhagen, Denmark) within 1 min to obtain the blood lactate value. The time point of the first blood lactate result≥4.0 mmol/L in the ICU was set as T0.T6 lactate was obtained within 1–9 h, T12 within 10–15 h, T24 within 16–27 h, T48 within 28–51 h, T72 lactate was obtained within 52–75 h after enrollment. At each time point, the closest to the specified time was taken. For example, T6 had two lactate values (4 h and 7 h respectively), the results of 7 h were taken. Demographic data, basic organ function, hemodynamic indicators and blood glucose levels at T0 and 6, 12, 24, 48, and 72 h after T0, cumulative clinical treatment conditions at different time points (fluid balance, doses of vasoactive and inotropic drugs, and amount of blood transfusion), and the final patient outcome were collected. The primary endpoint was all-cause mortality. The lactate kinetics at different time points were defined as follows: lactate kinetics_TX_ = (lactate_T0_–lactate_TX_)/lactate_T0_ × 100%. Regarding the sequence parameters, only parameters for which data were available for all time points were collected.

### Statistical analysis

Data analyses were performed using SAS statistical software (version 9.4; SAS Institute Inc., Cary, NC, USA). Continuous variables are expressed as the mean ± standard deviation. Variables with a normal distribution were compared using an independent sample t test. Data with an abnormal distribution are expressed as the median (interquartile range) and were compared using the Mann-Whitney U test. A two-side value of *P* < 0.05 indicated a significant difference. Receiver operating characteristic (ROC) curves of lactate kinetics at different time points were constructed, and the area under the ROC curve (AUC) for predicting all-cause mortality was calculated. Based on the maximum value (j) (i = sensitivity+specificity− 1) of Youden’s index, the best cutoff values for the above variables were confirmed. Factors (including clinical treatment conditions) associated with lactate kinetic targets at time points T6-T24 were screened using univariate and multivariate analyses. The clinical phenotypes of lactate kinetics were classified according to whether the lactate kinetic goals at 6 h, 12 h, and 24 h were attained. Logistic regression analyses were performed with death as the outcome to assess the odds ratios for different clinical phenotypes of lactate kinetics.

## Results

A total of 3298 patients, with 10,949 lactate measurements, were selected for this study. There were 1695 male patients, accounting for 51.3% of the enrolled patients. The average Acute Physiology and Chronic Health Evaluation (APACHE) II score was 17.56 ± 8.49 points, and the average Sequential Organ Failure Assessment (SOFA) score was 8.29 ± 4.33 points. A total of 402 patients died, and the mortality rate was 12.2%. The detailed baseline data are shown in Table 1.

**Table 1:** Demographic data of hyperlactatemia patients

Number of patients	3298
Sex (male, patients/%)	1695 (51.3)
Age (years)	56.58 ± 16.26
Department	
Emergency department (patients/%)	14 (0.43)
Internal medicine department (patients/%)	318 (9.64)
Surgical department (patients/%)	2535 (76.86)
Other hospital	431 (13.07)
Major disease	
Circulatory (patients/%)	646 (19.6%)
Respiratory (patients/%)	302 (9.2%)
Digestive (patients/%)	763 (23.1%)
Nervous system (patients/%)	189 (5.7%)
Endocrine (patients%)	214 (6.5%)
Immunological (patients/%)	81 (2.5%)
Kidney (patients/%)	119 (3.6%)
Bone (patients/%)	208 (6.3%)
Blood patients/%)	37 (1.1%)
Other (patients/%)	739 (22.4%)
APACHE II	17.56 ± 8.49
SOFA	8.29 ± 4.33
Baseline circulation	
CVP (mmHg)	9.17 ± 3.79
HR (bpm)	97.49 ± 20.97
SBP (mmHg)	135.22 ± 25.89
DBP (mmHg)	70.90 ± 14.55
MAP (mmHg)	92.69 ± 18.36
ScvO_2_ (%)	75.27 ± 11.37
Pcv-aCO_2_ (mmHg)	5.85 ± 3.43
Lac (mmol/L)	6.22 ± 3.15
Glu (mmol/L)	11.56 ± 3.83

*APACHE II* Acute Physiology and Chronic Health Evaluation, *SOFA* sequential organ failure assessment, *CVP* central venous pressure, *HR* heart rate, *SBP* systolic blood pressure, *DBP* diastolic blood pressure, *MAP* mean arterial pressure, *ScvO*_*2*_ central venous oxygen saturation, *Pcv-aCO*_*2*_ central venous-to-arterial blood carbon dioxide partial pressure difference, *Lac* lactate, *Glu* blood glucose

**Fig. 1:**
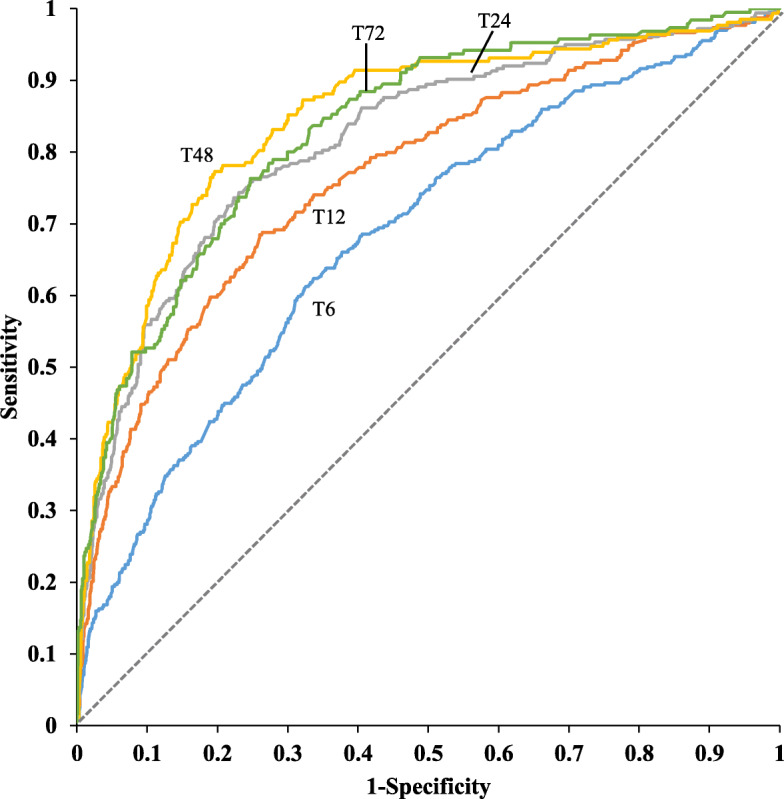
ROC curves of lactate kinetics at different timepoints for all-cause mortality. The lactate kinetics value at 6 h was 21%, and at 12 h, 24 h, 48 h, and 72 h, the lactate kinetics values were 40, 57, 66, and 72%, respectively. The area under the ROC curve of lactate kinetics at 6 h, 12 h, 24 h, 48 h, and 72 h for all-cause mortality were 0.684, 0.768, 0.818, 0.848, 0.831

Regarding hemodynamic indicators, central venous pressure (CVP), heart rate (HR), and lactate showed a gradual decreasing trend, with significant differences over time (Table 2). The lactate kinetics cutoff values for different time points are shown in Table 3 and Fig. 1. The lactate kinetics value at 6 h was 21%, and at 12 h, 24 h, 48 h, and 72 h, the lactate kinetics values were 40%, 57%, 66%, and 72%, respectively. The obtained cutoff values for T6, T12, and T24 lactate kinetics in this study were used to define target achievement when these cutoff values were met. Analyses were performed using unachieved targets as the outcome. Therefore, the APACHE II score, SOFA score, HR, and blood glucose were risk factors for goal achievement at different timepoints (Supplementary Table. S1).

**Table 2:** Hemodynamic indicators at different time points

	n	T0	T6	T12	T24	T48	T72	*P*
CVP	549	9.75 ± 3.8	9.7 ± 3.15	9.64 ± 2.91	9.41 ± 2.74^$^	8.96 ± 2.83^&^	8.27 ± 3.11^%^	< 0.0001
HR	1104	102.49 ± 20.65	100.75 ± 19.11*	99.37 ± 18.03^#^	98.57 ± 17.15^$^	95.71 ± 17.43^&^	92.35 ± 16.34^%^	< 0.0001
SBP	966	132.36 ± 24.49	130.04 ± 19.23*	131.65 ± 19.26	131.09 ± 20.06	132.7 ± 20.4	133.17 ± 20.93	0.001
DBP	965	68.63 ± 14.43	68.44 ± 12.06	68.79 ± 11.73	68.7 ± 12.7	69.26 ± 12.36	68.76 ± 12.04	0.5346
MAP	963	89.68 ± 17.46	88.2 ± 12.68*	88.86 ± 12.38	88.99 ± 13.21	89.96 ± 13.41	89.9 ± 14.08	0.0079
ScvO_2_	576	75.15 ± 11.68	74.53 ± 9.24	74.8 ± 8.7	74.21 ± 8.86	73.45 ± 9.26^&^	72.76 ± 9.42^%^	< 0.0001
Pcv-aCO_2_	695	5.59 ± 3.41	5.53 ± 3.02	5.09 ± 2.75^#^	4.72 ± 2.74^$^	5 ± 3.17^&^	5.17 ± 3.2^%^	< 0.0001
Lac	1179	6.89 ± 3.44	5.56 ± 3.93*	3.86 ± 3.33^#^	2.56 ± 2.38^$^	1.98 ± 2.27^&^	1.83 ± 2.6^%^	< 0.0001

*significant difference between T6 and T0; ^#^significant difference between T12 and T0; ^$^significant difference between T24 and T0; ^&^significant difference between T48 and T0; ^%^significant difference between T72 and T0; *P* < 0.05. *CVP* central venous pressure, *HR* heart rate, *SBP* systolic blood pressure, *DBP* diastolic blood pressure, *MAP* mean arterial pressure, *ScvO*_*2*_ central venous oxygen saturation, *Pcv-aCO*_*2*_ central venous-to-arterial blood carbon dioxide partial pressure difference, *Lac* lactate

**Table 3:** Cutoff values for lactate kinetics at different timepoints for all-cause mortality

Lactate kinetics	Best cutoff point	Sensitivity	Specificity	Youden’s index	AUC
T6	0.21	0.624	0.665	0.2894	0.684
T12	0.40	0.685	0.742	0.4271	0.768
T24	0.57	0.737	0.779	0.5161	0.818
T48	0.66	0.774	0.806	0.5801	0.848
T72	0.72	0.763	0.753	0.5165	0.831

Stratification was performed based on an APACHE II score of < 15 or ≥ 15 to further compare factors that influence achievement of lactate kinetics targets at different timepoints. The results showed that for patients with severe disease conditions (APACHE II score ≥ 15), the positive fluid balance and the norepinephrine dose for patients in the group that achieved lactate kinetics targets were significantly lower than those in the group that did not achieve the targets (Supplementary Table. S2).

A total of 1919 patients with complete lactate kinetics data within 24 h were divided into achieved and unachieved groups using the best cutoff point for the ROC curve for achievement of the target within 24 h, and their clinical phenotype groups were plotted using the assigned values. All-cause mortality was used as the outcome. Based on the patten of the timepoint achievements, eight clinical phenotypes were proposed (Table 4). Analyses of the influencing factors showed that when the goals at all timepoints were unachieved, the odds ratios of death increased by 4.39-fold (clinical phenotype VIII). When the lactate kinetics targets at 6 h were attained and at those at 12 and 24 h were not attained (clinical phenotype IV) or when the 24 h lactate kinetics target was not attained (clinical phenotype II), the odds ratios increased (Supplementary Table. S3).

**Table 4:** Lactate metabolism within 24 h (clinical phenotype groups based on whether the target was achieved or unachieved)

T6	T12	T24	Given value in the model	Number of patients (a total of 1919 cases)
Achieved	Achieved	Achieved	Clinical phenotype 1 (ref)	806
		Unachieved	Clinical phenotype 2	89
	Unachieved	Achieved	Clinical phenotype 3	74
		Unachieved	Clinical phenotype 4	79
Unachieved	Achieved	Achieved	Clinical phenotype 5	298
		Unachieved	Clinical phenotype 6	38
	Unachieved	Achieved	Clinical phenotype 7	223
		Unachieved	Clinical phenotype 8	312

## Discussion

This study retrospectively analyzed changes in the lactate kinetics of patients with hyperlactatemia and showed that lactate kinetics at 6 h, 12 h, 24 h, 48 h, and 72 h were 21, 40, 57, 66, and 72%, respectively. Using these values as standards, their predictive value for patient death gradually increased (AUC 0.684–0.848). Examination of the factors that influenced achieving 6 h, 12 h, and 24 h lactate kinetics targets showed that the APACHE II score, SOFA score, HR, and blood glucose were independent risk factors atthe time points that we measured. These results suggest that disease severity and the level of organ dysfunction affect the ability to achieve lactate kinetics targets. After stratification using the APACHE II score, the results showed that in critically ill patients (APACHE II ≥15), appropriate fluid balance and norepinephrine doses were beneficial for achieving lactate kinetics targets, whereas excessive positive fluid balance and large norepinephrine doses were harmful. Additionally, the effects of continuously achieving lactate kinetics targets on the prognosis were further analyzed and classified into eight clinical phenotypes. The results showed clinical phenotype VIII (T6, T12, and T24 targets were unachieved) had the higher odds ratio of patient death (OR = 4.39;95%CI 2.4–8.03). Even when the lactate kinetics target was achieved at 6 h but not at the following timepoints (clinical phenotype IV and II), the odds ratio still increased (OR = 4.2;95%CI 1.69–10.48 and OR = 5.27;95%CI 2.33–11.88, respectively).

Although some studies have explored the relationship between lactate kinetics and the prognosis of critically ill patients, some key issues, such as (1) the optimal cutoff value of lactate kinetics at different times and (2) the appropriate duration of monitoring lactate kinetics, remain unclear. To solve these problems, we first reviewed and analyzed the optimal cutoff value for prognosis at different timepoints. The results showed that the optimal cutoff values corresponding to these time points increased gradually. The EMShockNet investigators reported noninferiority in terms of reduction in hospital mortality among the group with lactate kinetics greater than 10% at 6 h and in the group with ScvO_2_ ≥ 70% at 6 h (17% vs 23%) [11]. Walker et al. reported in a retrospective study that resuscitation within 6 h and lactate kinetics of 36% could predict the prognoses of patients with sepsis [12].Masyuk et al. reported that lactate kinetics_T24h_ ≤ 19% was associated with increased ICU mortality (15% vs 43%; OR 4.11) [13]. In addition to the specific cutoff value differences, our results are consistent with previous studies because, on the one hand, the sample size of these studies is different;on the other hand, the prognosis and lactate kinetics of critically ill patients are closely related to disease heterogeneityand treatment differences in different centers [14, 15]. From the lessons learned from the failure of studies on supernormal goal-oriented therapy in the last century, we realized the importance of setting reasonable resuscitation goals [16, 17]. In fact, recovery of organ function, tissue perfusion, and even cell function during resuscitation requires time. Reasonable lactate kinetic goals can both produce the internal driving force to promote resuscitation and meet the physiological needs of the body to avoid excessive therapy caused by inappropriate and excessively high goals.

For patients with hyperlactatemia, how long should we monitor the lactate kinetics? Hernandez et al. [18] confirmed that only 52% of septic shock patients had normal blood lactate levels within 24 h. In a study by Maryna et al., for patients with lactate kinetics less than 19%, the average lactate level for the first 24 h was 5.25 mmol/L, and the average for the second 24 h was 6.43 mmol/L. Even in patients with lactate kinetics greater than 19%, the average lactate level for the first 24 h was 5.10 mmol/L, and the average for the second 24 h was 2.47 mmol/L. Thus, even after 24 h of resuscitation, a large number of patients still have hyperlactatemia and hypoperfusion. Therefore, monitoring 6 h, 12 h, or 24 h lactate kinetics alone is not sufficient to guide the entire process of resuscitation therapy. Based on the above reasons, our retrospective analysis of previous patients determined lactate kinetics cutoff values at five time points, from 6 h to 72 h. In addition, with the passage of time, the lactate kinetics gradually increased, and the ability to predict the survival rate of patients was also more evident.

Our study further examined the risk factors that influence whether the lactate kinetics at each time point reach these cutoff values. Various factors affect the achievement of lactate kinetics targets in clinical practice. Disease severity and the level of organ dysfunction are important components from our dataset. As representatives of these two aspects, the APACHE II score and SOFA score both show direct influences on achieving lactate kinetics goals, indicating that they are still reliable and indispensable evaluation tools for critically ill patients. Furthermore, additional attention should be paid to reductions in stress responses in critically ill patients, and the HR is a sensitive indicator of stress in the body. Many studies in recent years have confirmed that sepsis patients obtained excellent effects after applying β-receptor blockers to control the ventricular rate [19–21] to reduce stress responses. For cardiogenic shock patients, reduction in the ventricular rate can improve ventricular diastolic function to further improve the ventricular stroke volume and overall cardiac efficiency, which is beneficial for improving tissue perfusion and the prognosis [22, 23]. In our study, HR was an independent risk factor for achieving lactate kinetics targets from 6 h to 24 h, which again confirms that the influence of HR on the treatment of critically ill patients requires attention. High blood glucose is also a presentation of stress responses in critically ill patients. One recent study showed that a high blood glucose level was an independent risk factor for in-hospital death of cardiogenic shock patients [24]. Another study showed that, for both cardiogenic shock and septic shock, hyperlactatemia was mainly caused by an increase in lactate production and that the increase in lactate production was usually accompanied by high blood glucose and an increase in glucose turnover, indicating that the latter had great impacts on lactate metabolism [25]. Our study also showed that blood glucose affected achieving lactate kinetics targets, suggesting that blood glucose control should be a focus during shock resuscitation.

After the lactate kinetics targets were confirmed, we evaluated the effects of clinical resuscitation measures on dynamic attainment. The results showed that for patients with critical illness (APACHE II score ≥ 15 points), there was less positive fluid balance in achieved lactate kinetics targets group. Although previous studies have confirmed that conservative fluid management strategy can improve the prognosis of patients in the post-resuscitation phase (after hemodynamic stabilization) [26], it still needs to be confirmed whether precise fluid resuscitation strategy is beneficial to patients during resuscitation. Previous studies showed that the application of norepinephrine to increase the blood pressure of septic shock patients to 85 mmHg did not benefit tissue oxygen metabolism, skin capillary blood flow, and urine output [27]. Our study found that when the blood pressure levels of the two groups were similar after resuscitation from the clinical view, the dosage of norepinephrine was lower in achieved lactate kinetic target group, which was more likely associated with more profound vasoplegia. in the non-achieved target group.

By observing the change in the trajectory of lactate kinetics while reaching the cutoff values, we confirmed that the group that continuously reached the cutoff values had an obviously better outcome than that of the group that reached the cutoff values at any time point. According to these dynamic changesin lactate kinetics, we proposed clinical phenotypes of lactate kinetics to identify the most critical points and the phenotype for the best prognosis. The effect of the previously reported 6 h lactate kinetics attainment rate on the prognosis was not as good as that of the 12 h and 24 h attainment rates, which was partially consistent with results from a previous study [28]. Clinically, the phenotype of lactate changes can be used to determine the patient prognosis. These results suggested that, under limited resource conditions, greater focus should be placed on achieving 12 and 24 h lactate kinetics goals rather than 6 h lactate kinetics goals. Additionally, attaining high lactate kinetics goals might require more fluid infusion during treatment, the application of more inotropic drugs to increase cardiac output, and setting a higher arterial pressure, which might cause harm [16, 17]. The phenotype theory based on the lactate kinetics cutoff values at different timepoints represents a milestone for the entire resuscitation process; thus, the goals during resuscitation are clearer, and insufficient or excessive resuscitation can be avoided.

Our study had some limitations. First, this study was a retrospective, single-center study. The confirmed lactate kinetics cutoff values at different timepoints lack broad representation. Factors such as differences in therapeutic strategies at different medical institutions (such as blood transfusion and cardiotonic therapy) and timeliness of treatment might influence the determination of cutoff values. Therefore, a multicenter study with a clear and unified treatment plan is needed for further verification. Second, this study targeted all critically ill patients. In the future, the lactate kinetics of patients with certain diseases, such as septic shock and cardiogenic shock, can be explored according to disease classification in order to more accurately guide clinical treatment. Third, this study included a 72 h period, but some patients did not remain in the ICU for this length of time due to death and transfer out. Over time, fewer patients were included in the analysis. Therefore, we can only include all of the parameters that we can obtain, and we cannot rule out actual factors that affect the data. Fourth, the production and metabolism of lactate is a complex process. In addition to hemodynamics, it may also be affected by stress, liver function and some treatment measures (such as CRRT). These factors need to be considered in the follow-up study to reveal the law of lactate kinetics more comprehensively. Fifth, In the study design, we defined the time point of the first blood lactate result ≥4.0 mmol / L in the ICU as T0, rather than the time of onset of the patient. Therefore, the level of lactate in T0 can not accurately reflect the situation of patients at the onset of disease. However, we mainly focus on the follow-up lactate dynamics. The setting of the initial time point may be in conditional (may be limited), but probably not very significant.

## Conclusion

Stepwise recovery of lactate kinetics is an important resuscitation target for patients with severe hyperlactatemia. The cutoff values for lactate kinetics at 6 h, 12 h, 24 h, 48 h, and 72 h that influenced patient prognosis were 21, 40, 57, 66, and 72%, respectively. The APACHE II score, SOFA score, HR, and blood glucose are independent risk factors that influenced achievement lactate kinetic targets. When the lactate clearance rate is high, additional fluid support and vasoactive drugs are not needed. Clinical phenotypes of stepwise lactate kinetics are proposed, which may contribute to assessmentof the prognosis. Although our conclusions are based on a large sample size, the conclusions of this study need to be supported by prospective multicenter studies in the future.

## Supplementary Information

**Additional file 1: Supplementary Table S1**. Influencing factors of the group that did not achieve lactate kinetics targets at timepoints T6-T24. **Supplementary Table S2**. Factors that influenced achievement of lactate kinetics targets at different timepoints. **Supplementary Table S3**. Effects of continuously achieving lactate kinetics targets and related indicators on mortality

## Data Availability

The datasets during and/or analysed during the current study available from the corresponding author on reasonable request.

## Abbreviations

ICU: Intensive care unit
ROC: Receiver operating characteristic
AUC: The area under the ROC curve
APACHE II: Acute Physiology and Chronic Health Evaluation II
SOFA: Sequential Organ Failure Assessment
CVP: Central venous pressure
HR: Heart rate
SBP: Systolic blood pressure
DBP: Diastolic blood pressure
MAP: Mean arterial pressure
ScvO2: Central venous oxygen saturation
Pcv-aCO2: Central venous-to-arterial blood carbon dioxide partial pressure difference
Lac: Lactate
Glu: Blood glucose
CRRT: Continue renal replacement therapy
