# Supplementary material for: Stepwise lactate kinetics in critically ill patients: prognostic, influencing factors, and clinical phenotype
Source: BMC Anesthesiol. 2021 Mar 19;21:86. doi: 10.1186/s12871-021-01293-x (PMC7977296; doi:10.1186/s12871-021-01293-x)
Supplement: Supplementary file 1 — Additional file 1: Supplementary Table S1. Influencing factors of the group that did not achieve lactate kinetics targets at timepoints T6-T24. Supplementary Table S2. Factors that influenced achievement of lactate kinetics targets at different timepoints. Supplementary Table S3. Effects of continuously achieving lactate kinetics targets and related indicators on mortality [file 12871_2021_1293_MOESM1_ESM.docx]

**Supplementary material**

**Supplementary tables**

Supplementary Table S1 - Influencing factors of the group that did not achieve lactate kinetics targets at timepoints T6-T24

|  |  | Univariate | | Multivariate | |
| --- | --- | --- | --- | --- | --- |
|  |  | OR (95% CI) | P | OR (95% CI) | P |
|  | Age (every 10 years vs. <20 years) | 1.05 (1, 1.1) | 0.0604 | 1.05 (0.98, 1.12) | 0.1877 |
|  | Sex (male vs. female) | 1.07 (0.93, 1.24) | 0.3421 | 0.83 (0.67, 1.02) | 0.076 |
|  | APACHE II score (T0, every 5 points vs. <5 points) | 1.35 (1.29, 1.42) | <0.0001 | 1.23 (1.14, 1.32) | <0.0001 |
|  | SOFA score (T0, every 5 points vs. <5 points) | 1.66 (1.52, 1.82) | <0.0001 | 1.21 (1.05, 1.4) | 0.0103 |
| T6 | Heart rate (T6, every 10 bpm vs. <60 bpm) | 1.22 (1.17, 1.27) | <0.0001 | 1.11 (1.04, 1.18) | 0.0017 |
|  | Invasive average blood pressure (T6, every 10 mmHg vs. <60 mmHg) | 0.93 (0.88, 0.99) | 0.0136 | 0.97 (0.9, 1.05) | 0.4918 |
|  | SvO_2_ (T6) | 0.99 (0.98, 1) | 0.1163 | 1.01 (1, 1.03) | 0.0236 |
|  | Pcv-aCO_2_ (T6) | 1.04 (1.02, 1.07) | 0.0026 | 1.07 (1.03, 1.11) | 0.0004 |
|  | Blood glucose (T6) | 1.15 (1.12, 1.18) | <0.0001 | 1.12 (1.09, 1.15) | <0.0001 |
| T12 | Age (every 10 years vs. <20 years) | 1.04 (0.98, 1.09) | 0.2061 | 1.04 (0.96, 1.12) | 0.3768 |
|  | Sex (male vs. female) | 1.06 (0.9, 1.26) | 0.4693 | 0.98 (0.77, 1.24) | 0.8532 |
|  | APACHE II score (T0, every 5 points vs. <5 points) | 1.48 (1.39, 1.56) | <0.0001 | 1.27 (1.17, 1.38) | <0.0001 |
|  | SOFA score (T0, every 5 points vs. <5 points) | 2.1 (1.88, 2.35) | <0.0001 | 1.48 (1.24, 1.76) | <0.0001 |
|  | Heart rate (T12, every 10 bpm vs. <60 bpm) | 1.26 (1.2, 1.33) | <0.0001 | 1.12 (1.04, 1.21) | 0.0029 |
|  | Invasive average blood pressure (T6, every 10 mmHg vs. <60 mmHg) | 0.95 (0.89, 1.02) | 0.1325 | 1.02 (0.93, 1.12) | 0.6621 |
|  | SvO_2_ (T12) | 0.99 (0.98, 1) | 0.0164 | 1.01 (1, 1.03) | 0.0864 |
|  | Pcv-aCO_2_ (T12) | 1.02 (0.99, 1.05) | 0.2596 | 1.05 (1, 1.09) | 0.0365 |
|  | Blood glucose (T12) | 1.19 (1.15, 1.23) | <0.0001 | 1.15 (1.1, 1.2) | <0.0001 |
| T24 | Age (every 10 years vs. <20 years) | 1.1 (1.04, 1.17) | 0.0016 | 1.04 (0.94, 1.14) | 0.4542 |
|  | Sex (male vs. female) | 1.09 (0.9, 1.32) | 0.3677 | 0.86 (0.65, 1.14) | 0.2994 |
|  | APACHE II score (T24, every 5 points vs. <5 points) | 1.51 (1.41, 1.61) | <0.0001 | 1.27 (1.15, 1.39) | <.0001 |
|  | SOFA score (T24, every 5 points vs. <5 points) | 1.91 (1.66, 2.21) | <0.0001 | 1.78 (1.44, 2.19) | <.0001 |
|  | Heart rate (T24, every 10 bpm vs. <60 bpm) | 1.25 (1.17, 1.34) | <0.0001 | 1.15 (1.04, 1.26) | 0.0041 |
|  | Invasive average blood pressure (T24, every 10 mmHg vs. <60 mmHg) | 1.02 (0.94, 1.1) | 0.6136 | 1.09 (0.98, 1.21) | 0.1225 |
|  | Pcv-aCO_2_ (T24) | 0.99 (0.98, 1) | 0.0833 | 1 (0.99, 1.02) | 0.8153 |
|  | Pcv-aCO_2_ (T24) | 1.01 (0.97, 1.04) | 0.7441 | 0.99 (0.94, 1.05) | 0.8095 |
|  | Blood glucose (T24) | 1.1 (1.06, 1.14) | <0.0001 | 1.06 (1, 1.13) | 0.0413 |

Supplementary Table S2 - Factors that influenced achievement of lactate kinetics targets at different timepoints

|  | Unachieved | | Achieved | | P* |
| --- | --- | --- | --- | --- | --- |
|  | n | Median (P25, P75) | n | Median (P25, P75) |  |
| T6 |  |  |  |  |  |
| **APACHE II score T0<15** |  |  |  |  |  |
| Fluid balance | 352 | 218.3 (-92.8, 626.58) | 907 | 140 (-204, 562) | 0.0450 |
| Norepinephrine dose | 2 | 8.5 (6, 11) | 12 | 6.5 (3, 16) | 0.9270 |
| Adrenaline dose | 0 |  | 0 |  |  |
| Milrinone dose | 0 |  | 6 | 6600 (4000, 10000) |  |
| Dobutamine dose | 0 |  | 4 | 30 (10, 75) |  |
| **APACHE II score T0≥15** |  |  |  |  |  |
| Fluid balance | 679 | 704.4 (86, 1341.85) | 886 | 320.43 (-129.8, 879.6) | <0.0001 |
| Norepinephrine dose | 156 | 24 (12, 71) | 60 | 12.5 (4, 25.5) | <0.0001 |
| Adrenaline dose | 36 | 11.5 (4, 20.5) | 7 | 6 (2, 6) | 0.2613 |
| Milrinone dose | 24 | 5700 (2000, 8250) | 15 | 3000 (1200, 7200) | 0.3615 |
| Dobutamine dose | 10 | 50 (30, 110) | 9 | 40 (10, 60) | 0.2838 |
| T12 |  |  |  |  |  |
| **APACHE II score T0<15** |  |  |  |  |  |
| Fluid balance | 184 | 271.55 (-226.85, 728.05) | 779 | 166.9 (-339.65, 684.3) | 0.1744 |
| Norepinephrine dose | 4 | 12.5 (7.5, 18) | 12 | 6 (2.5, 30) | 0.6708 |
| Adrenaline dose | 0 |  | 0 |  |  |
| Milrinone dose | 1 | 11000 (11000, 11000) | 6 | 9500 (3000, 14400) | 0.8026 |
| Dobutamine dose | 1 | 180 (180, 180) | 5 | 40 (10, 70) | 0.5525 |
| **APACHE II score T0≥15** |  |  |  |  |  |
| Fluid balance | 499 | 874.95 (-15.95, 2121.7) | 816 | 386.35 (-259.9, 1099.14) | <0.0001 |
| Norepinephrine dose | 157 | 37 (18, 88) | 57 | 17 (7, 32) | <0.0001 |
| Adrenaline dose | 34 | 15 (7, 42) | 8 | 7 (1, 16.5) | 0.0628 |
| Milrinone dose | 24 | 4400 (1000, 10000) | 22 | 9300 (2000, 13000) | 0.1122 |
| Dobutamine dose | 8 | 65 (25, 185) | 9 | 80 (70, 240) | 0.3586 |
| T24 |  |  |  |  |  |
| **APACHE II score T24<15** |  |  |  |  |  |
| Fluid balance | 168 | 177.3 (-815.41, 1180.73) | 694 | -173.78 (-1120.7, 800.3) | 0.0127 |
| Norepinephrine dose | 24 | 18.5 (3, 44) | 34 | 11.5 (4, 24) | 0.4766 |
| Adrenaline dose | 0 |  | 0 |  |  |
| Milrinone dose | 2 | 10500 (10000, 11000) | 14 | 6900 (3000, 14400) | 0.4249 |
| Dobutamine dose | 4 | 200 (50, 530) | 4 | 40 (10, 70) | 0.3065 |
| **APACHEII score T24≥15** |  |  |  |  |  |
| Fluid balance | 322 | 748.95 (-736.2, 2539.35) | 484 | 227.43 (-896.83, 1396.85) | 0.0005 |
| Norepinephrine dose | 128 | 49 (20.5, 108.5) | 62 | 17.5 (7, 44) | <0.0001 |
| Adrenaline dose | 25 | 29 (8, 60) | 13 | 6 (1, 7) | 0.0007 |
| Milrinone dose | 18 | 4500 (2000, 18000) | 24 | 13500 (1600, 23000) | 0.4372 |
| Dobutamine dose | 5 | 70 (30, 210) | 12 | 165 (80, 240) | 0.3968 |

Supplementary Table S3 - Effects of continuously achieving lactate kinetics targets and related indicators on mortality

|  | Multivariate model* | |
| --- | --- | --- |
|  | OR (95% CI) | P |
| Clinical phenotype 2 (vs. Clinical phenotype 1) | 5.27 (2.33, 11.88) | <0.0001 |
| Clinical phenotype 3 (vs. Clinical phenotype 1) | 2.52 (0.88, 7.16) | 0.0839 |
| Clinical phenotype 4 (vs. Clinical phenotype 1) | 4.2 (1.69, 10.48) | 0.0021 |
| Clinical phenotype 5 (vs. Clinical phenotype 1) | 0.37 (0.13, 1.08) | 0.0697 |
| Clinical phenotype 6 (vs. Clinical phenotype 1) | 1.78 (0.38, 8.39) | 0.465 |
| Clinical phenotype 7 (vs. Clinical phenotype 1) | 0.76 (0.33, 1.8) | 0.538 |
| Clinical phenotype 8 (vs. Clinical phenotype 1) | 4.39 (2.4, 8.03) | <0.0001 |
| Age (every 10 years vs. <20 years) | 1.03 (0.89, 1.2) | 0.6546 |
| Sex (male vs. female) | 0.6 (0.38, 0.93) | 0.0215 |
| APACHE II score (T24, every 5 points vs. <5 points) | 1.66 (1.44, 1.92) | <0.0001 |
| SOFA score (T24, every 5 points vs. <5 points) | 2.24 (1.56, 3.22) | <0.0001 |
| Heart rate (T24, every 10 bpm vs. <60 bpm) | 0.9 (0.78, 1.03) | 0.1253 |
| Invasive average blood pressure (T24, every 10 mmHg vs. <60 mmHg) | 0.8 (0.68, 0.94) | 0.0062 |
| SvO_2_ (T24,every 1%) | 0.98 (0.96, 1) | 0.0767 |
| Pcv-aCO_2_ (T24, every 1mmHg ) | 0.91 (0.84, 0.99) | 0.0273 |
| Blood glucose (T24, every 1mmol/L) | 1.02 (0.94, 1.11) | 0.5999 |
